# Radiomics of high-resolution computed tomography for the differentiation between cholesteatoma and middle ear inflammation: effects of post-reconstruction methods in a dual-center study

**DOI:** 10.1007/s00330-020-07564-4

**Published:** 2020-12-04

**Authors:** Christophe T. Arendt, Doris Leithner, Marius E. Mayerhoefer, Peter Gibbs, Christian Czerny, Christoph Arnoldner, Iris Burck, Martin Leinung, Yasemin Tanyildizi, Lukas Lenga, Simon S. Martin, Thomas J. Vogl, Ruediger E. Schernthaner

**Affiliations:** 1 Department of Diagnostic and Interventional Radiology, University Hospital Frankfurt, Frankfurt am Main, Germany; 2 Department of Radiology, Memorial Sloan Kettering Cancer Center, New York, NY USA; 3 Department of Biomedical Imaging and Image-guided Therapy, Division of General and Pediatric Radiology, Medical University of Vienna, Waehringer Guertel 18-20, 1090 Vienna, Austria; 4 Department of Otorhinolaryngology, Head Neck Surgery, Medical University of Vienna, Vienna, Austria; 5 Department of Otolaryngology, Head and Neck Surgery, University Hospital Frankfurt, Frankfurt am Main, Germany; 6 Department of Neuroradiology, University Medical Center of the Johannes Gutenberg-University, Mainz, Germany

**Keywords:** Tomography, X-ray computed, Temporal bone, Cholesteatoma, Otitis media, Retrospective studies

## Abstract

**Objectives:**

To evaluate the performance of radiomic features extracted from high-resolution computed tomography (HRCT) for the differentiation between cholesteatoma and middle ear inflammation (MEI), and to investigate the impact of post-reconstruction harmonization and data resampling.

**Methods:**

One hundred patients were included in this retrospective dual-center study: 48 with histology-proven cholesteatoma (center A: 23; center B: 25) and 52 with MEI (A: 27; B: 25). Radiomic features (co-occurrence and run-length matrix, absolute gradient, autoregressive model, Haar wavelet transform) were extracted from manually defined 2D-ROIs. The ten best features for lesion differentiation were selected using probability of error and average correlation coefficients. A multi-layer perceptron feed-forward artificial neural network (MLP-ANN) was used for radiomics-based classification, with histopathology serving as the reference standard (70% of cases for training, 30% for validation). The analysis was performed five times each on (a) unmodified data and on data that were (b) resampled to the same matrix size, and (c) corrected for acquisition protocol differences using ComBat harmonization.

**Results:**

Using unmodified data, the MLP-ANN classification yielded an overall median area under the receiver operating characteristic curve (AUC) of 0.78 (0.72–0.84). Using original data from center A and resampled data from center B, an overall median AUC of 0.88 (0.82–0.99) was yielded, while using ComBat harmonized data, an overall median AUC of 0.89 (0.79–0.92) was revealed.

**Conclusion:**

Radiomic features extracted from HRCT differentiate between cholesteatoma and MEI. When using multi-centric data obtained with differences in CT acquisition parameters, data resampling and ComBat post-reconstruction harmonization clearly improve radiomics-based lesion classification.

**Key Points:**

*• Unenhanced high-resolution CT coupled with radiomics analysis may be useful for the differentiation between cholesteatoma and middle ear inflammation.*

*• Pooling of data extracted from inhomogeneous CT datasets does not appear meaningful without further post-processing.*

*• When using multi-centric CT data obtained with differences in acquisition parameters, post-reconstruction harmonization and data resampling clearly improve radiomics-based soft-tissue differentiation.*

## Introduction

One of the most common diseases of the tympanic cavity besides middle ear inflammation (MEI) is cholesteatoma, which is an expanding growth consisting of the epithelium surrounded by inflammatory reaction [1]. Currently, diagnostic workup and preoperative planning of clinically suspected cholesteatoma or complicated MEI are mostly performed using high-resolution computed tomography (HRCT) of the temporal bone [2]. In both MEI, especially when chronic, and cholesteatoma, erosion of surrounding bony structures can occur and surgical intervention might be indicated. Although the presence of cholesteatoma is typically suggested by its location in the attic or sinus tympani [3], both entities can appear in different areas of the tympanic cavity, which limits their discrimination on HRCT. While diffusion-weighted imaging (DWI) may aid the diagnosis of cholesteatoma, inflammatory tissue and high protein fluid in MEI might lead to false positive results [4], whereas early and small cholesteatomas may be missed [5]. As a consequence, a final diagnosis can presently only be established by histopathologic analysis of surgically removed tissue. Accurate preoperative discrimination between both diseases is of high clinical importance as the therapy for cholesteatoma is usually surgery whereas chronic MEI can be treated conservatively in most cases [6]. In addition, preoperative information on the possible underlying disease is important for the surgical approach and technique to be chosen. Therefore, improved differentiation of cholesteatoma and MEI by means of imaging has without doubt a considerable clinical impact and efforts to do so are needed.

Radiomics is an emerging field in medical imaging that may potentially advance current clinical practice. Radiomics is based on the assumption that genetic, molecular, and biological properties of tissues are correlated with signal intensity patterns within medical images (i.e., spatial variations of gray-level values of pixels and voxels), such as CT and MRI. Notably, computed-assisted radiomic analyses can extract a multitude of so-called radiomic features (mathematical descriptors of image texture and shape) that the human eye cannot assess, let alone quantify [7]. Nevertheless, the use of multi-center data for radiomics analyses can be problematic, as differences in acquisition parameters are known to have a considerable effect on radiomic features [8]. A prerequisite for the widespread application of radiomics in clinical practice is the development and investigation of post-processing methods, as strictly homogeneous data acquisition between multiple centers is unlikely to be achieved [9].

The main goal of the present study was to determine whether radiomic features extracted from unenhanced HRCT can be used to differentiate between cholesteatoma and MEI—not just in a single-, but also in a dual-center setting. It was also of special interest to investigate the impact of post-reconstruction techniques in that regard. To our knowledge, the utility of radiomic signatures derived from cross-sectional imaging for the assessment of diseases of the temporal bone, including cholesteatoma and MEI, has not been investigated yet. We hypothesized that microstructural differences between the two types of soft tissue masses would lead to characteristic patterns in HRCT that can be quantified using radiomics analysis, and that post-processing could potentially improve its value.

## Material and methods

This retrospective dual-center study conforms to Health Insurance Portability and Accountability Act (HIPAA) guidelines and was approved by the local Institutional Review Boards/Ethics committees; informed consent was waived.

### Patient selection

In center A, a database search was performed for patients who underwent initial unenhanced HRCT for clinically suspected cholesteatoma between January 2017 and September 2018. In center B, a retrospective review of surgery log books of January 2006 to January 2010 was performed to identify patients that underwent surgery due to suspected cholesteatoma or MEI. Final confirmation of diagnosis was based on intra-operative histology in all cases. In both centers, exclusion criteria were former surgery of the middle ear, no pathological findings in HRCT, and lack of preoperative HRCT or post-operative histology. In both centers, patients were included consecutively; neither in children nor in adults, size or other morphological criteria of the lesions on HRCT were used for patient selection.

### Imaging protocol

In center A, all studies were performed on a 192-slice third-generation dual-source CT scanner (Somatom Force, Siemens Healthineers) operating at 120 kV and 150 mAs. Acquisition was performed in craniocaudal direction with a pitch of 0.85 and collimation of 0.4 mm. Automatic tube current modulation (Caredose 4D, Siemens Healthineers) was activated in all examinations. All data were reconstructed as axial, paracoronal, and parasagittal images with a matrix size of 512 × 512 voxels, a section thickness of 0.4 mm, and increment of 0.2 mm. An advanced modeled iterative reconstruction algorithm (Admire, Siemens Healthineers) was used at a strength level of 3 out of 5.

In center B, all patients underwent state-of-the-art HRCT on a 64-row multi-detector CT scanner (Brilliance 64, Philips Healthcare) at 140 kV and 200 mAs. Acquisition was performed in craniocaudal direction (pitch, 0.35; collimation, 0.63 mm). Axial, coronal, and sagittal image series were reconstructed with a matrix size of 768 × 768 voxels, slice thickness of 0.67 mm, and increment of 0.33 mm. Filtered-back projection reconstruction was used.

CT scans of younger children were conducted in sedation, if necessary, to facilitate diagnostic image quality and to avoid the necessity to repeat an acquisition.

### Image post-processing

Data resampling was performed by a radiologist (R.E.S), using DCMScale of the DCM Toolkit (https://support.dcmtk.org/docs/dcmscale.htm). Data from center B was resampled from a 768 × 768 to a 512 × 512 matrix (+Sxv 512) using DCMScale’s standard interpolation (+i 1) to match the matrix used in center A.

Post-reconstruction harmonization of data in terms of correction for acquisition protocol differences between the two centers/scanners was applied directly to radiomic feature values. For this study, the ComBat method was used [10]. ComBat harmonization was originally developed for genomic data and is based on the removal of the center effect on numerical values of extracted radiomic features.

### Image analysis and radiomics

**Fig. 1:**
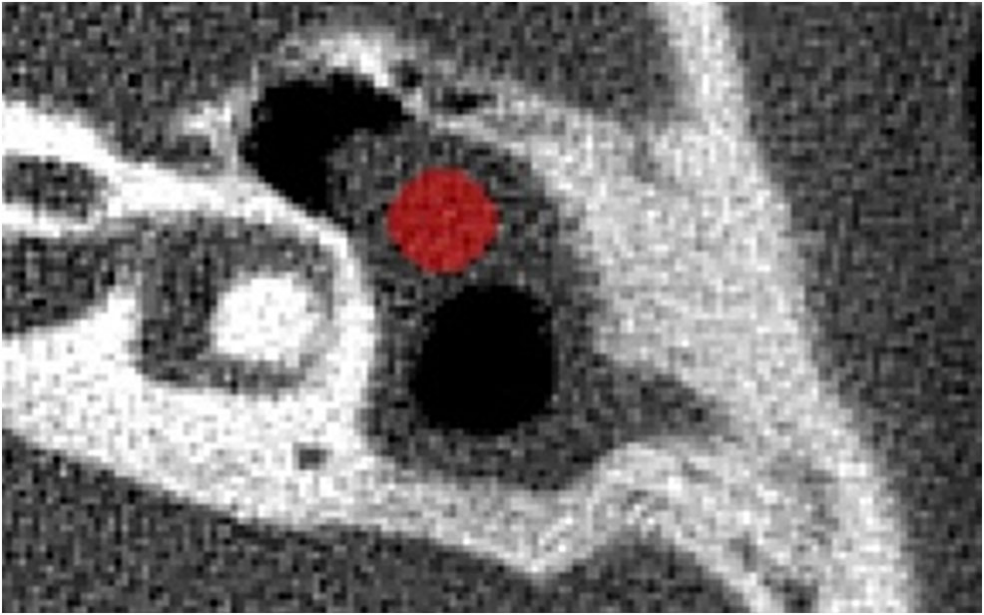
Manual region of interest placement for radiomics analysis in an 11-year-old boy with clinically suspected cholesteatoma in the left middle ear

Axial HRCT image series from both centers were used for radiomics analysis. For feature extraction, a semi-automatic approach was selected using publicly available software MaZda 4.6 (http://www.eletel.p.lodz.pl/programy/mazda). In all cases, two board-certified radiologists with 6 years of experience each in consensus chose one representative slice and placed a single 2D circular region of interest (> 4 mm^2^) in the center of the middle ear lesion (Fig. 1). A particular slice was chosen to capture the lesion in its largest extent while at the same time preserving adequate distance from surrounding anatomical structures. Gray-level normalization was applied in every region of interest to reduce potential influences of brightness and contrast variations on feature quantification [11], limiting dynamics to *μ* ± 3*σ* (*μ*, gray-level mean; *σ*, standard deviation) [12]. A multitude of radiomic features (*n* = 279 per lesion) from the following categories of radiomic features were extracted from the manually defined regions of interest: first-order histogram (HIS; *n* = 9; gray-level statistics such as percentiles), co-occurrence matrix (COM; *n* = 220; distribution of pixel pairs with predefined gray-level values and interpixel distances), run-length matrix (RUN; *n* = 20; distribution of runs of pixels with the same gray-level value), absolute gradient (GRA; *n* = 5; degree/abruptness of gray-level value changes between neighboring pixels), autoregressive model (ARM; *n* = 5; degree of gray-level randomness/regularity), and discrete Haar wavelet transform (WAV; *n* = 73; frequency content of an image at different scales). The full list of radiomic features that MaZda is capable of calculating can be found at http://www.eletel.p.lodz.pl/programy/mazda/download/FeaturerList.pdf.

### Statistical analysis and classification

The utility of HRCT radiomics for differentiation between cholesteatoma and MEI was evaluated in different datasets:

1. in unmodified single-center data (i.e., separate evaluation of data from the two centers);
2. in unmodified pooled data from the two centers;
3. in pooled data from the two centers that were resampled to the same matrix size; and
4. in pooled data from the two centers that were corrected for acquisition protocol differences using ComBat harmonization.

For all above analyses, feature selection was performed as a first step, to reduce the large number of features obtained initially to the most relevant feature sets based on mathematical criteria. For this study, POE + ACC (probability of error and average correlation) coefficients were used to select subsets of the 10 best radiomic features for differentiation between cholesteatoma and MEI. This method was chosen because in addition to identification of features that best distinguish between classes, it also addresses data redundancy (i.e., the correlation between individual radiomic features).

For the separate analyses of unmodified data from the two centers, linear discriminant analysis (LDA), which generates the most discriminating features based on the sets of 10 features identified in the previous step, was applied for further dimensionality reduction. This was done due to the lower number of samples per center, to avoid overfitting—i.e., producing overoptimistic estimates of classification performance. Overfitting may occur when the number of features considered is very high in comparison to the number of samples analyzed, because in this case, random correlations may be detected. For the same reason, a K-nearest neighbor (k-NN) approach with leave-one-out cross-validation was used for classification in these separate monocentric datasets; i.e., the model was trained using all patients of a center excluding one patient (*n* − 1) and tested on the held-out patient, to keep the number of samples high relative to the number of radiomic features; this process was repeated *n* times [12]. Rates of misclassified vectors yielded by the k-NN classifier were used to calculate accuracies, which served as the main outcome variable in these groups. LDA and k-NN classification was performed for data from centers A and B separately, and subsequently, for pooled data from both centers.

For pooled (unmodified or post-processed) data, a multi-layer perceptron feed-forward artificial neural network (MLP-ANN), which is based on a back-propagation learning algorithm, was used for radiomics-based classification. Contrary to the single-center analysis, the cohort was considered sufficiently large to use this more advanced technique. Here, 70% of cases were randomly assigned to the training dataset, and 30% to the validation dataset. Because the initial step taken by the neural network is a “guess” at the weights of the individual radiomic features—potentially leading to different classification results, the analysis was performed five times each, to provide a more robust/realistic estimate of classification performance. In addition, for each repetition of the classification step, patients were newly randomized and assigned to training or validation group. For MLP-ANN, a minimum of one hidden layer with a minimum of three neurons per layer was used. Areas under the receiver operating characteristic (ROC) curves (AUC) and classification accuracies for training and validation datasets were calculated. MLP-ANN was applied to the pooled unmodified data, the pooled ComBat harmonized data, and the pooled data that were resampled to the same matrix. For the latter, the regions of interest (ROIs) were drawn anew on resampled images to obtain radiomics features; POE + ACC feature selection was also repeated. Histopathology obtained from surgery specimens served as the standard of reference.

## Results

In center A, 18 out of the initial group of 68 patients were excluded due to former surgery of the middle ear (*n* = 15) or lack of pathological findings in HRCT (*n* = 3). All remaining 23 patients received surgery after HRCT and were histologically diagnosed with cholesteatoma. Another 27 patients with histological or clinical diagnosis of MEI were used as a control group. Mean patient age was 33.1 ± 19.9 (range, 6–78 years); 38 patients were male.

In center B, 69 consecutive patients who underwent surgery due to cholesteatoma were retrospectively included. Out of these patients, 44 had to be excluded due to lack of preoperative HRCT (*n* = 42) or post-operative histology (*n* = 2). Another 25 patients with MEI were retrospectively selected as a control group. Mean age was 39.5 ± 22.3 years (range, 5–81 years); 24 patients were male.

**Fig. 2:**
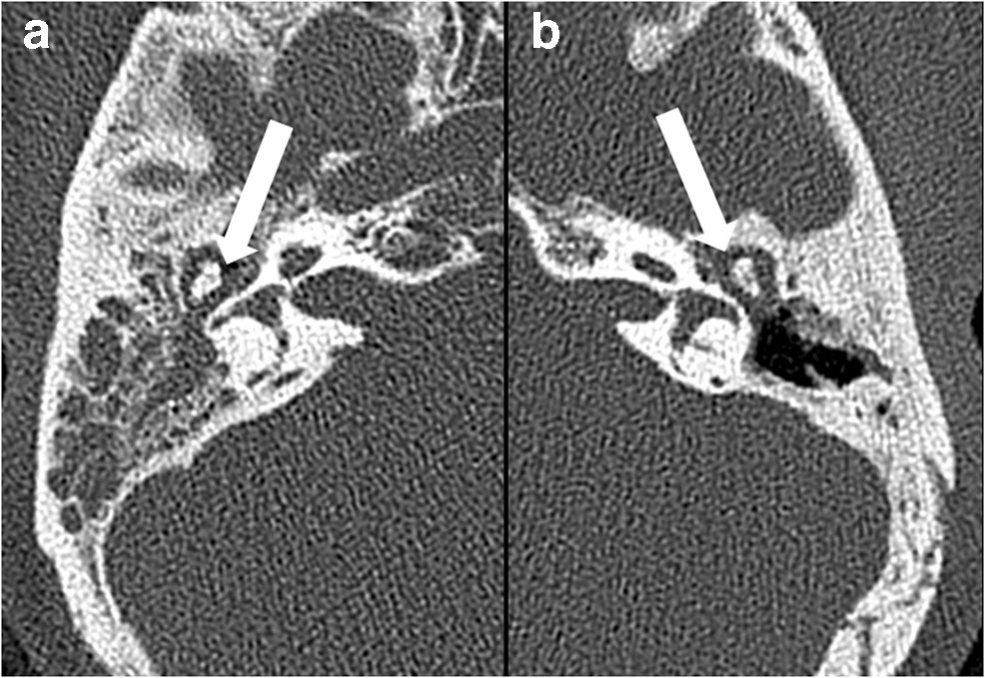
Left: unenhanced high-resolution computed tomography (HRCT) image of a 54-year-old female patient with middle ear inflammation (MEI) in the right tympanic cavity (**a**). Right: unenhanced HRCT image of a 19-year-old male patient with a cholesteatoma in the left tympanic cavity (**b**). Both show soft tissue in the middle ear without bone destruction. Radiomics characteristics derived from unenhanced HRCT-differentiated MEI from cholesteatoma with an overall median area under the receiver operating characteristic curve (AUC) of 0.78 (separate accuracies: center A, 66%; center B, 84%) in our patient collective. Post-processing in terms of data resampling and harmonization yielded overall median AUCs of 0.88, and 0.89, respectively

Thus, 100 patients (center A, 50 patients, 23 with cholesteatoma, 27 with MEI; center B, 50 patients, 25 with cholesteatoma, 25 with MEI) were included in this study (Fig. 2).

### Unmodified data

Separately for data from center A, 33/50 cases (accuracy, 66%; misclassified: 9 cholesteatoma, 8 MEI) were correctly classified, whereas separately for data from center B, 42/50 cases (accuracy, 84%; misclassified: 4 cholesteatoma, 4 MEI) were correctly classified.

Using unmodified pooled data from the two centers, MLP-ANN classification yielded an overall median AUC of 0.78 (0.72–0.84), with median accuracies of 71.4% (65.7–85.7%) in the training and 66.7% (63.3–71%) in the validation datasets (Fig. 2).

### Pooled post-processed data

Using original data from center A with a 512 × 512 matrix and data from center B resampled to the same matrix, MLP-ANN classification revealed an overall median AUC of 0.88 (0.82–0.99) with median accuracies of 83.8% (79.2–95.9%) in the training and 76.9% (71.4–85.2%) in the validation datasets.

**Fig. 3:**
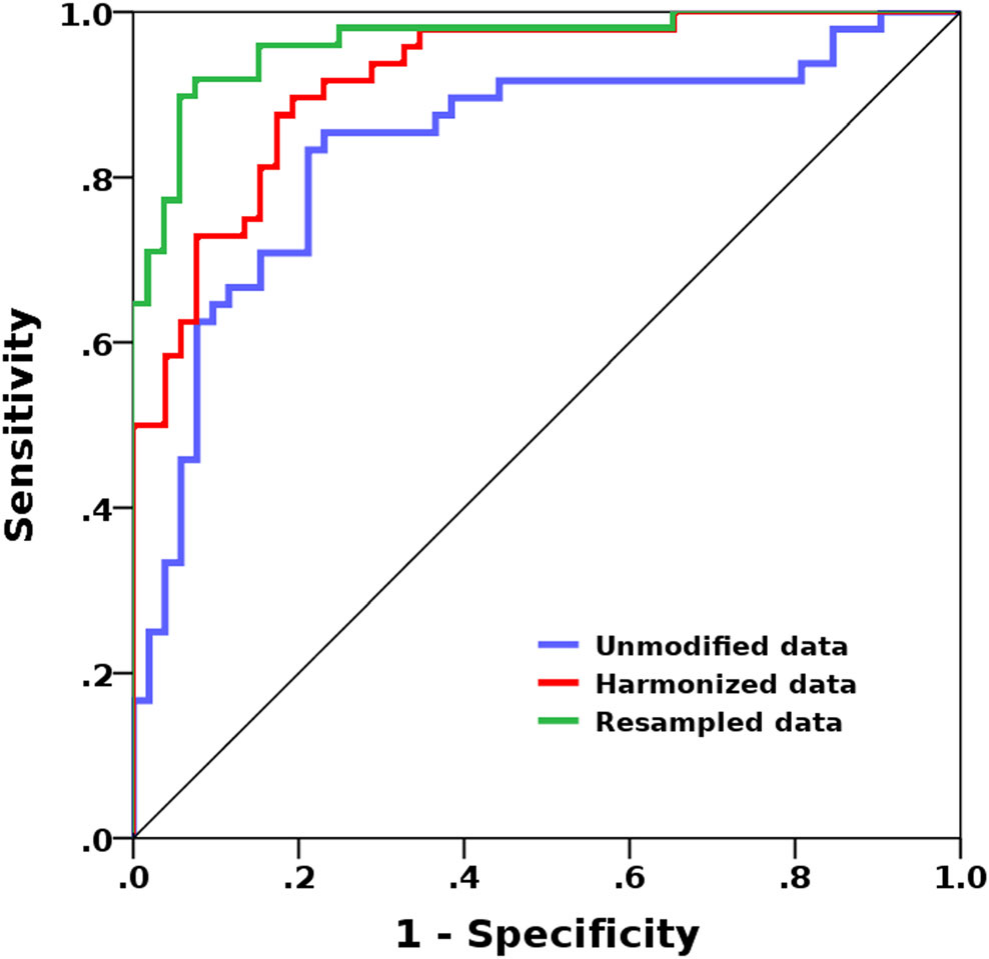
Results from the receiver operating characteristic (ROC) curve analysis for the radiomics-based separation of patients with middle ear inflammation and cholesteatoma using dual-center data. An initial area under the curve (AUC) of 0.78 could be improved to 0.88 and 0.89 using post-processing in terms of data resampling and harmonization

Using pooled ComBat harmonized data, MLP-ANN classification yielded an overall median AUC of 0.89 (0.79–0.92), with median accuracies of 82.9% (68.6–88.6%) in the training and 73.3% (60.0–76.7%) in the validation datasets (Fig. 3).

## Discussion

In this dual-center study, we evaluated the utility of unenhanced HRCT radiomic signatures for the non-invasive discrimination of cholesteatoma and MEI, and additionally investigated the impact of two post-reconstruction methods in that regard. Our results indicate that radiomic analyses of pooled inhomogeneous data from multiple centers should not be performed without further post-processing. Both ComBat harmonization and resampling to identical matrix size similarly improve radiomics-based lesion classification. Hence, both methods seem to be valuable post-reconstruction techniques for multi-center radiomics analyses, and need to be investigated further. After additional validation, unenhanced HRCT coupled with radiomics analysis may be a useful tool for the image-based differentiation between cholesteatoma and MEI.

Radiomic analyses performed on various types of imaging data have yielded promising results for the separation of different tumor entities throughout the body [13–15]. An accurate identification of cholesteatoma among other soft tissue masses is especially relevant due to its locally aggressive nature, possibly leading to permanent damage to surrounding anatomical structures, and high risk of recurrence [16]. The diagnosis of cholesteatoma in preoperative HRCT mostly relies on typical lesion location and presence of bony erosion; however, this approach is known to be limited [17]. Previous attempts to differentiate cholesteatoma and MEI have been made. For instance, Trojanowska et al suggested a potential usefulness of post-contrast CT for the detection of cholesteatoma in 17 patients [18], while De Foer et al emphasized the value of DWI and T1-weighted sequences in that regard [19]. Also Profant et al recommended non-echo planar imaging DWI as a valid method for the diagnosis and follow-up of cholesteatoma [20]. Shie et al proposed a feature-based classification system for otoscopic diagnosis of MEI, including, but not limited to, HIS features [21]. In a study including 91 patients, Lee et al concluded that measurements of Hounsfield units (HU)—i.e., HIS features—are not sufficient to distinguish between the two diseases [22]. Park et al, however, found HU values to be significantly different between cholesteatoma and inflammatory tissue in 82 patients [23]. Our results show that among the variety of radiomic features, HIS features did not seem to be relevant for radiomics-based lesion classification, as opposed to COM (co-occurrence matrix; based on the distribution of pixel pairs with predefined gray-level intensities), RUN (run-length matrix; based on sequences of pixels with the same gray-level intensity), and WAV (Haar wavelet transform; based on image decomposition for capturing details and edges in different directions across an image) features (Table 1) [24]. In particular, COM features such as angular second moment, which measures the gray-level homogeneity or order of gray-level intensities, and contrast, which emphasizes gray-level intensity differences between the two pixels of a pixel pair, dominated the list of relevant features. Unlike these “true” radiomic features that reflect spatial variations of gray-level intensities across an ROI, HIS features are statistical descriptors of signal intensities.

**Table 1:** Selected feature sets for radiomics-based separation of middle ear inflammation and cholesteatoma using pooled dual-center data

Unmodified and harmonized	Resampled
WavEnLL_s-2^1^	S(2,0)SumVarnc^2^
S(1,1)InvDfMom^2^	S(0,5)DifVarnc^2^
S(2,0)SumOfSqs^2^	S(0,1)SumAverg^2^
S(1,-1)SumAverg^2^	S(0,3)InvDfMom^2^
S(3,0)Correlat^2^	S(0,2)Correlat^2^
S(0,4)Contrast^2^	S(2,2)Correlat^2^
S(0,3)AngScMom^2^	S(2,-2)Correlat^2^
S(3,3)Correlat^2^	45dgr_LngREmph^3^
S(2,0)InvDfMom^2^	S(3,-3)Contrast^2^
45dgr_GLevNonU^3^	WavEnLL_s-2^2^

^1^Discrete Haar wavelet transform: WavEnLL_s-2, wavelet transform energy after bi-directional low-pass filtering

^2^Co-occurrence matrix: *AngScMom*, angular second moment; *Correlat*, correlation; *SumAverg*, sum average; *SumOfSqs*, sum of squares; *SumVarnc*, sum variance; *InvDfMom*, inverse difference moment; values in parentheses reflect interpixel distances and coordinates/directions for pixel pairs

^3^Run-length matrix: 45dgr_GLevNonU, gray-level non-uniformity calculated in 45° direction; 45dgr_LngREmph, long-run emphasis calculated in 45° direction

Previous studies have shown that radiomics features are affected by image acquisition parameters such as slice thickness and reconstruction algorithm. In a recent systematic review of 41 studies, Traverso et al observed the strongest effect of acquisition parameter variations on reproducibility for spatial resolution, followed by scan duration and method of reconstruction [25]. In general, first-order and shape features were found to be more stable than texture features, which capture lesion heterogeneity [26, 27]. As a result, prior investigators have yielded variable results for the utility of radiomics signatures for lesion differentiation when heterogeneous data in terms of scanner and imaging protocol was used [28]. Likewise, pooling of multi-center data does not seem meaningful without further post-processing in the present study, achieving an overall median AUC of only 0.78 for the separation of MEI and cholesteatoma. In view of the literature and the fact that, in our study, HRCT data from center B, which were obtained with higher slice thickness, but a larger matrix size, yielded better results than data from center A, we hypothesize that for future radiomics studies on this or similar topics, HRCT protocols should put particular emphasis on high in-plane resolution.

Post-reconstruction methods such as data resampling (i.e., preimage analysis) and data harmonization (i.e., post-image analysis) are currently being investigated as potential solutions to this known problem in the field of radiomics. Harmonization relies on mathematical post-processing to remove effects of image acquisition parameter variations on already-existing radiomics features. The currently popular application ComBat has been found to be beneficial for positron emission tomography (PET) radiomics in previous studies [10, 29]. A very recent study has also confirmed the effectiveness of ComBat for correction of CT radiomic data [30]. Resampling of voxel size is considered to be another promising strategy to correct for effects due to differences in spatial resolution between scanners [31]. Our results suggest that both data resampling and harmonization may be helpful applications for multi-center radiomics studies involving large amounts of heterogeneous data, removing the impact of acquisition differences while preserving pathophysiological information. With an increase in AUC from 0.78 to 0.88 and 0.89, data resampling and post-reconstruction harmonization yielded similarly good to excellent results in terms of accuracy for the discrimination of cholesteatoma and MEI. Notably, resampling and ComBat harmonization are applied at very different levels in the analytic process. Resampling is applied before, and ComBat harmonization after radiomic feature extraction, meaning that one technique operates directly on the images, and the other on the numerical feature values. Given these fundamental differences, it is very difficult to say why resampling performed slightly better in our present study, or whether this observation can be generalized—to our knowledge, this is the first study to compare both techniques in the same sample. It is, however, apparent that, to yield meaningful results, future radiomics studies require high-quality data, obtained with homogeneous imaging protocols and/or corrected for image acquisition differences when this cannot be achieved [32].

This study has some limitations beyond its retrospective design, which need to be acknowledged. First, our patient cohort is relatively small; however, neither class imbalance nor overfitting which are common pitfalls in radiomics research, should have an impact on our results, as classes and rates of misclassified cases are balanced throughout the study. Second, while methods such as k-NN, LDA, and MLP-ANN are well established [33, 34], more advanced convolutional neural networks with larger numbers of hidden layers are considered the gold standard in the field of machine learning. Nonetheless, a model should be chosen according to cohort size to avoid overfitting. Third, for the sake of consistency, ROIs were drawn in round shape, manually and on one slice only, thus possibly omitting characteristic portions of the lesions in terms of gray-level heterogeneity that would have been captured using, for instance, mesh volumes of interest. Clearly, future studies would therefore need to include confirmatory 3D analyses, preferably with automatic lesion segmentation. Fourth, since different matrix sizes were used routinely at each study site and raw data were not available due to retrospective design, resampling of reconstructed data was necessary. For the same reason, we were unable to evaluate the influence of the number of iterations on radiomic features. Finally, a direct comparison of HRCT radiomics with DWI should be performed in a future trial to investigate the interchangeability of HRCT radiomics and DWI for the differentiation of cholesteatoma and middle ear inflammation. The combination of HRCT with DWI data in future studies might even further improve the relevance of radiomics analysis, as different aspects of tissue biology such as heterogeneity and diffusivity could be taken into account.

In conclusion, our data indicate that radiomic features derived from unenhanced HRCT may be useful for the differentiation between cholesteatoma and MEI. However, pooling of data extracted from inhomogeneous CT datasets does not appear to be meaningful without further post-processing. When using multi-centric data obtained with differences in CT acquisition parameters, ComBat post-reconstruction harmonization and data resampling clearly improve radiomics-based lesion classification. Unenhanced HRCT may be a valuable addition to DWI for soft tissue differentiation between cholesteatoma and MEI when radiomic features are utilized.

## Abbreviations

COM: Co-occurrence matrix
HIS: First-order histogram
HRCT: High-resolution CT
k-NN: K-nearest neighbor
LDA: Linear discriminant analysis
MEI: Middle ear inflammation
MLP-ANN: Multi-layer perceptron feed-forward artificial neural network
POE + ACC: Probability of error and average correlation
